# SARS-CoV-2 historical global testing and genomic variability

**DOI:** 10.1186/s12879-022-07147-2

**Published:** 2022-04-01

**Authors:** Halim Tannous, Shadi Akiki, Rasha E. Boulos, Charlene El Khoury Eid, Ghadi El Hasbani, Lea Maria Khoueiry, Lynn El Khoury, Rawan Tohme, Rim Moussa, Georges Khazen

**Affiliations:** 1 Gilbert and Rose Mary Chagoury School of Medicine, Lebanese American University, Byblos, Lebanon; 2 Independent Researcher, Montclair, USA; 3 Computer Science and Mathematics Department, School of Arts and Science, Lebanese American University, Byblos, Lebanon

**Keywords:** SARS-CoV-2, Covid19 testing, Covid19 genomic variability, SARS-CoV-2 testing, SARS-CoV-2 genomic variability

## Abstract

The world has been dealing with the SARS-COV-2 pandemic since December 2019 and a lot of effort has focused on tracking the spread of the virus by gathering information regarding testing statistics and generating viral genomic sequences. Unfortunately, there is neither a single comprehensive resource with global historical testing data nor a centralized database with summary statistics of the identified genomic variants. We merged different pre-aggregated historical testing data and complemented them with our manually extracted ones, which consist of 6852 historical test statistics from 76 countries/states unreported in any other dataset, at the date of submission, making our dataset the most comprehensive to date. We also analyzed all publicly deposited SARS-CoV-2 genomic sequences in GISAID and annotated their variants. Both datasets can be accessed through our interactive dashboard which also provides important insights on different outbreak trends across countries and states. The dashboard is available at https://bioinfo.lau.edu.lb/gkhazen/covid19. A daily updated version of the datasets can be downloaded from github.com/KhazenLab/covid19-data.

**Supplementary Information:**

The online version contains supplementary material available at 10.1186/s12879-022-07147-2.

## Background

A novel coronavirus, SARS-Cov-2 first appeared in Wuhan, China in December 2019 and the World Health Organization declared it as a global pandemic on March 11, 2020. Since the start of this pandemic, a lot of effort has focused on gathering different outbreak metrics such as confirmed-cases, fatalities and testing statistics. We can list here most notably: Our World in Data (OWID) [1], Johns Hopkins University (JHU) [2], Covid Tracking Project (CTP) (covidtracking.com), Wikipedia (wikipedia.org/wiki/COVID-19_testing) and Worldometers (worldometers.info/coronavirus).

Unfortunately, the majority of the platforms sharing historical testing statistics lack substantial amounts of data. For instance, CTP focuses only on the number of tests per state in the United States of America, while OWID aggregates data, by country only and not states, from multiple sources. However, at the date of submission, OWID, which is considered one of the most comprehensive platforms for testing data, covered only 94 countries out of the 195 (ourworldindata.org/coronavirus-testing) and had only partial testing data for some countries.

Consequently, we manually extracted historical testing data (LAU manual subset) from different sources, and combined it with these pre-aggregated datasets.

Although different national mitigation measures can lead to different outbreak metrics, it is crucial to look at the genomic variability of the virus across different spatiotemporal points. Mutations are known to occur both naturally and frequently in viruses, but might result in an increased pathogenicity, virulence and even resistance [3]. In fact, the analysis of the genomic variability of SARS-CoV-2 revealed a high occurrence in structural genes [4, 5]. The majority of these studies were conducted on a relatively small number of samples. Our belief is that a more comprehensive and integrative genomic variability study will help better understand the differences in the virus outbreak severity. Therefore, we regularly analyze the SARS-CoV-2 genomic sequences deposited in GISAID [6] and share the identified variants as well as their consequence annotations with the scientific community.

In this paper, we present both a testing numbers dataset and a genomic variability dataset to help the scientific community and the decision makers in their effort to fight against Sars-CoV-2. In addition, we present an analysis of the testing and confirmed cases data to elaborate on the trends of Sars-CoV-2 for each country. The rest of the paper is structured as follows. The “Construction and content” section presents the data collection, validation and processing for both the testing and genomic variability datasets and presents the dashboard developed to highlight the datasets. The “Utility and Discussion” section highlights the utility of our testing data analysis. The final section “Conclusion” concludes the paper.

## Construction and content

### Global testing data

#### Testing data collection

We initially collect the number of cases and fatalities from JHU (github.com/CSSEGISandData/COVID-19). This represents our basis for data collection and specifies the countries we will be gathering testing data for. We assemble historical testing data from 4 major sources: OWID, CTP, Worldometers and Wikipedia. We store a daily snapshot of Worldometers and Wikipedia pages, since they are overridden daily. Countries unreported in these 4 sources are then identified and ranked in descending order based on their respective cumulative numbers of cases from JHU, and assigned for manual collection. We then traceback the historical testing numbers from the 4 sources and identify the days with missing data.

We try to collect as many missing historical testing data points as possible from different sources, which are listed in Additional file 1: Table S1 for each country/state. The table consists of 7 fields: Country/State, First Data Point, Last Data Point, Language, Data Type, Test Type reported and the Source reference.

The “First Data Point” field indicates the date of the first data point that we manually collected.

The “Last Data Point” field indicates the date of the last data point that we manually collected. Usually, if other sources (such as OWID) start covering a country, we stop collecting data points manually unless to fill critical gaps in OWID data.

The “Language” field indicates the language using which the collected data is represented. Any language other than English/ French and Arabic are translated using the Google chrome extension tool, or using other translation websites (itools.com/tool/google-translate-web-page-translator).

The “Data Type” field indicates the format of the data (API: application programming interface, Infographic: uploaded data that gets overridden daily, Daily reports, News reports, Graphs and Machine readable datasets).

The “Test Type Reported” field indicates the method used for testing PCR, serological or unspecified.

Some of these listed sources show historical data, while others report the testing numbers on the current day and override the webpage information daily. As a solution, we use the wayback machine (archive.org/web/) to navigate back to older versions.

After manually collecting testing data, we merge all the testing datasets together, including our LAU manual subset, into one comprehensive historical dataset.

#### Testing data validation

We run the data collection pipeline on a daily basis and re-validate the data based on the following criteria:

1. If there are inconsistencies between the number of tests and the reported cases in one of the resources, then only testing data higher than the number of reported cases is considered.
2. In the case of overlap, daily testing data are chosen from only one source and prioritized as follows: OWID, CTP, LAU manual subset, Wikipedia, Worldometers. We put Worldometers last because they do not provide a date for their collected numbers.

It is worthwhile noting that with every new run, the sources used might update their previous historical data which might result in new redundancies. Whenever this situation is presented, criteria 1 and 2 will resolve any inconsistency or duplication in the data.

We validate the gathered data to eliminate illogical test numbers. We look for three main issues in the gathered data: decreasing numbers of cumulative confirmed cases, decreasing numbers of cumulative tests, daily number of daily tests less than daily cases. If these cases are presented for a certain country/state at a certain date, we eliminate the conflicting testing number. Until 2020-09-01, the number of testing data points dropped is 4742, of which 2075 were due to decreasing numbers of cumulative tests and 2667 due to daily tests less than daily cases. The number of confirmed cases dropped from the JHU dataset is 128 due to decreasing cumulative cases.

**Fig. 1:**
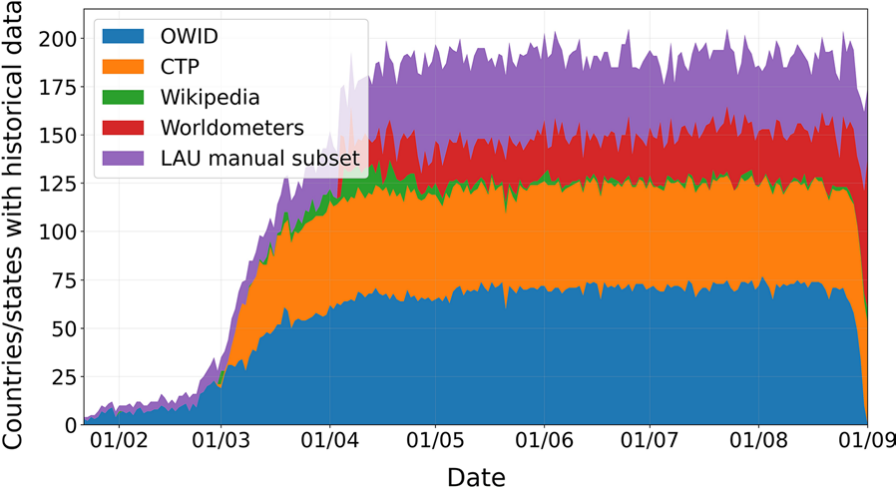
Number of countries/states covered per day by each data source in our dataset

Contribution to the comprehensive dataset by source (at the date of submission) is presented in Table 1. Our manually extracted data accounts for 21% (6852) of our dataset and covers 74 country/state pairs, of which 49 have less than 25% date coverage in any of the four initial sources at the date of submission (Fig. 1). Finally, we validate our gathered data and perform data cleaning to eliminate illogical entries.

**Table 1:** Dataset contribution by source

	OWID	CTP	LAU Manual Subset	Worldometers	Wikipedia
Data points	12,291	9261	6852	3647	635
(%)	38	28	21	11	2

#### Testing data post-processing

We post-process the data in order to properly visualize it on our dashboard and to conduct our testing analysis. The post-processing involves the replacement of missing testing numbers; which can be either on the last collection date or on a previous date, and a spike easing step. For the last collection date we use an extension approach and for the previous dates we use an interpolation approach. These 2 steps are conducted for visual purposes, and will also be used when computing our metrics and when conducting our testing analysis. They do not contribute to the numbers represented in Additional file 1: Table S1 and Table 1.

Extension approach: replaces the missing testing data on the last collection date. The number of tests are extended in order to account for the global cumulative number of tests. For instance, when our dataset reaches 2020-05-18, France’s testing data points stop at 1,384,633 cumulative tests on 2020-05-08. Hence without extending these data points to reach 2020-05-18, this would lead to a decrease in the number of global cumulative tests on 2020-05-09 by the same value. To extend the latest missing numbers of tests, we compute the daily positive cases for the missing days, and add these values to the latest available number of tests. This results in the extension of the numbers of tests using only confirmed cases. This, however, assumes that the number of negative tests per day is null.

Interpolation approach: replaces missing testing data between known test number values. There are some constraints here that should be taken into consideration. For instance, the daily number of tests should not be lower than the daily positive cases. For this reason, we use Eq. (3) below to replace the missing testing data points. Assuming that two data points are separated by missing cumulative test number values, and that the two data points have known cumulative tests and cumulative case number (all numbers below represent cumulative numbers) we compute the following:

1 $$Translation\,factor \left( {Tf} \right) = tests_{before} - cases_{before}$$

2 $$Scaling\,factor \left( {Sf} \right) = \frac{{tests_{after} - cases_{after} + Tf}}{{cases_{after} + Tf}}$$

3 $$Tests\left[ i \right] = floor\left[ {\left( {cases\left[ i \right] + Tf} \right)*\left( {1 + Sf} \right)*\frac{i}{N - 1}} \right]$$

where *N* is the number of days between the first and last available test point, and *i* represents the number of days for which the test number is estimated (*i* varies between *0* and *N* − 1).

An example is given in Table 2. In this example, linear interpolation causes no issue when comparing linearly-interpolated cumulative tests with cumulative cases. However, the linearly-interpolated daily tests present an issue on day 3 and day 4 where daily tests are lower than daily cases. Applying our algorithm, we achieved estimated numbers of tests that do not violate any of the aforementioned conditions.

$$Translation\,factor \left( {Tf} \right) = 10 - 2 = 8$$

$$Scaling\,factor \left( {Sf} \right) = \frac{50 - 40 - 8}{{40 + 8}} = \frac{2}{48}$$

**Fig. 2:**
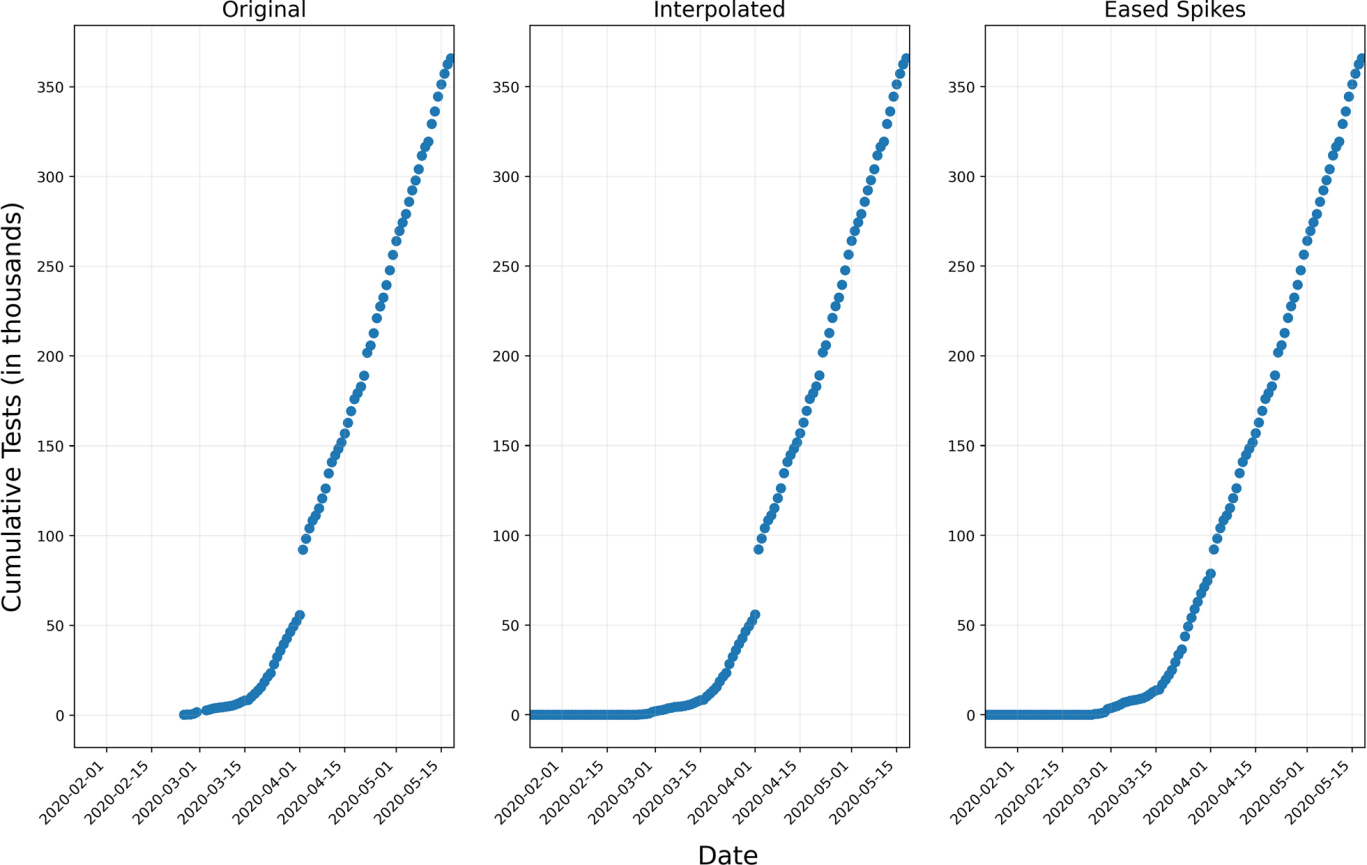
Number of cumulative tests in Austria: left is the original data, middle is interpolated, and right is after spike easing

Spike easing approach: Another aspect of our data post-processing concentrates on cases where countries spontaneously add new testing information from additional laboratories without correcting the full cumulative history, which could lead to sudden spikes in the testing data. To account for these discontinuities, we smooth out large spikes into smaller jumps, spread over the previous few days. For example, Austria’s cumulative tests have the following progression from 2020-03-31 until 2020-04-02: 5200, 5600, 9200 (Fig. 2). Clearly, the jump to 9200 on 2020-04-02 is not in line with the daily numbers seen before, so the 3600 (9200–5600) are distributed linearly over all days from the date of the first case, i.e. the beginning of March until April 2, thus raising the curve before the jump and avoiding the spike. This spike easing method yields less false alarms when calculating the significance of change in the number of positive cases described in the methods section.

**Table 2:** Interpolation example

	Day 1	Day 2	Day 3	Day 4
Cumulative cases	2	3	20	40
Daily cases	2	1	17	20
Cumulative tests	10	NA	NA	50
Linearly-interpolated cumulative tests	10	23	36	50
Linearly-interpolated daily tests	10	13	13^A^	14^a^
Our method cumulative tests	10	11	28	50
Our method daily tests	10	1	17	22

^a^Violation of the condition daily tests > daily cases

The extension, interpolation and spike easing approaches are represented, in white, to the end user on the dashboard, and are used to analyse our testing metrics.

### SARS-CoV-2 genomic sequences

#### Genomics variability data collection

The viral genomic sequences are downloaded from GISAID and processed on a weekly basis. Only complete or near complete genomes (length > 29,000) are compared to the first reported sequence from Wuhan, China (Accession number: EPI_ISL_402125) using Mummer version 3.1 [7] with default parameters. The identified variants are functionally annotated using snpEff [8] with default parameters using NC_045512.2 as a reference. Annotations of the reference genome are downloaded from GenBank [9] (NCBI Reference Sequence:NC_045512.2).

#### Genomic variability data validation

The genomic variability pipeline is run on a weekly basis to detect mutations and get their consequence annotations. 92 354 sequences from 156 countries or territories were analyzed until the date of submission. The analysis resulted in 166 sequences without any mutation (SNPs, insertions/deletions, breakpoint, relocation, translocation, inversion). A total of 25 223 unique SNPs was identified. While some occur frequently in the dataset others only appear one time, but the majority correspond to missense (57.77%) and synonymous (29.46%). Consistently, similarly to previous studies we observe a higher diversity in the genes N, S, ORF3A and ORF8 [4, 5, 10, 11]. Interestingly, those same genes present the highest missenses mutations relative to the synonymous ones.

The most common variant, 23403A > G-(D614G), observed 73,070 times and commonly found in the USA, Australia and European countries, is the same as the one described as a potential drift and a threat for vaccine development [5]. Interestingly, this variant did not occur alone, it co-exists with 241C > T (in 5′ UTR), 3037C > T-(F105F) and 14408C > T-(P323L). We also observed a striking co-occurrence phenomena of 28144T > C-(L84S) and 8782C > T-(S57S), similar to the observation of other study [12].

### Dashboard

The dashboard is developed using ESRI (esri.com), Shiny (shiny.rstudio.com) and Bokeh (bokeh.org), and is divided into two sections: “Global Testing” and “Genomic Variability”. The Global Testing section includes four parts:

Interactive map: provides a global view of the cumulative tests/million and confirmed-cases/tests as well as two distribution plots of the daily confirmed-cases and number of tests.

Summary statistics: provides a graphical representation of the different daily and cumulative outbreak metrics both globally and by country/state.

Comparative statistics: compares the metrics listed in the “Metrics extraction” section below between a maximum of 10 different countries/states.

Trends: summarizes the rate of change of testing and confirmed-cases per country and provides the expected minimum and maximum number of cases expected to detect a significant change in the number of confirmed-cases per country. Details about this information is presented in the “Testing and confirmed-cases analysis” section below.

The Genomic Variability page summarizes the average number of mutations per 1 kb in each gene, within each country and sample. It also provides their relative frequencies per gene and consequence types.

## Utility and discussion

### Testing data analysis

#### Metrics extraction

We compute six daily and six cumulative statistics for each country/state: total tests, negative tests, confirmed-cases, tests/million, tests/confirmed-cases and confirmed-cases/tests.

#### Testing and confirmed-cases analysis

In order to compute a country’s testing and confirmed cases trends, we start by calculating the last week’s rate of change in testing and confirmed-cases for each country using a 7-day moving average. We disregard countries that have not updated their testing data over the past 3 days. These numbers would give a first impression at a country’s strategy when dealing with SARS-CoV-2 (e.g. is the country’s testing ratio higher than the confirmed cases ratio over the past week). However, this alone does not help us conclude on the country’s situation.

**Fig. 3:**
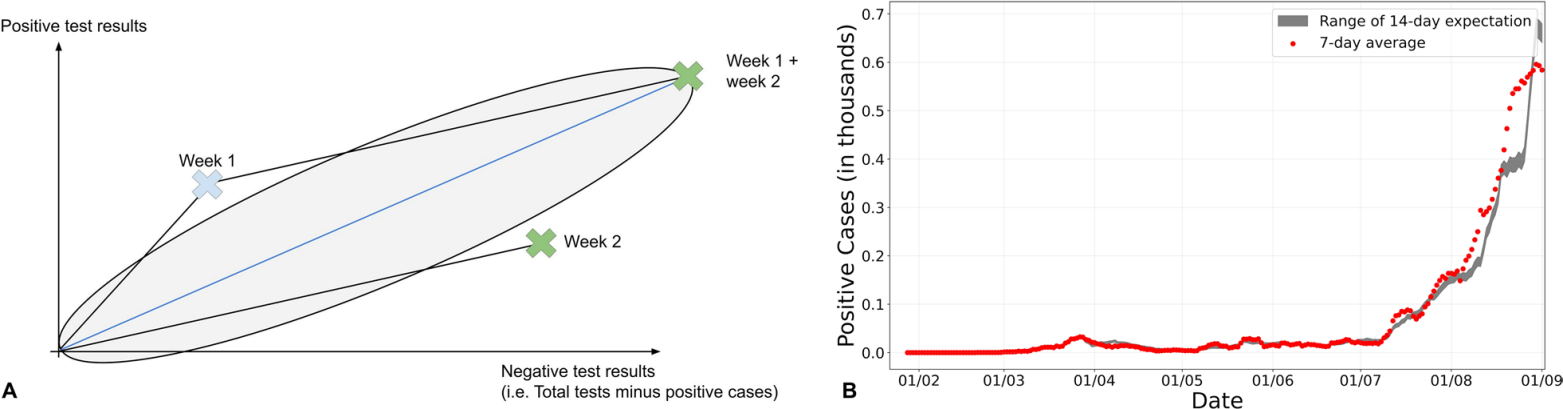
**A** An illustration of the graphical method. This specific example shows the case where the observed number of positive cases for week 2 (current week) is significantly less than that from week 1 (previous week). **B** 7-day Moving average of the positive cases (red) vs the range of the 14-day expectation (grey)

For this reason, we also compute the 7-day moving average of the number of cases for the last 14 days and use the chi-squared test to check if there is a significant increase or decrease in the number of cases between the current (Week 2) and previous week (Week 1). Additionally, using the graphical method from Bolles et al. [13], we compute the minimum and maximum number of 7-day average of cases needed per day to detect either a significant increase or decrease in the number of cases with a 95% confidence level. The Bolles et al. graphical method yields an ellipse with the x-axis representing the number of negative test results, and the y-axis representing the number of positive test results. The ellipse’s boundary represents the minimum and maximum number of positive cases allowed given a certain number of negative cases such that there is no significant change in the number of cases at a p-value of 5%, Fig. 3A illustrates the cumulative tests and cases interpretation. In fact, if the observed number is outside the ellipse boundary, the change in the number of cases is then considered to be significant, above the boundary indicates a significant increase while below it indicates a significant decrease.

Figure 3B shows an example of this analysis, represented to the end-user on our dashboard. This particular example is that of ''Lebanon''. The figure highlights the 7-day average positive cases (red dots) recorded during the period starting from 2020-01-22 until 2020-09-01, compared to the 14-day expectation range generated as a result of the chi-square test (grey range).

### Dataset utility in the fight against SARS-COV-2

The dataset will prove to be a very useful tool in the fight against SARS-CoV-2. First, the testing dataset is the most comprehensive dataset pertaining to historical testing numbers of SASRS-CoV-2, available online for researchers to use. In addition, we present a validated dataset that does not contain erroneous elements that could affect future analysis. Moreover, this dataset also presents interpolated data points, for missing testing data, that are logically interpolated.

On the other hand, the genomics dataset presents an important advantage as it directly presents ready to use data, helping researchers skip the tedious tasks of preparing their dataset before using it.

Finally, the major advantage presented is the regular dynamic update that we provide to our datasets. This would allow researchers to implement their codes and analysis without the need to update the data constantly.

## Conclusion

In this paper, we present our comprehensive testing and genomic variability datasets for SARS-CoV-2. The datasets presented are validated, post-processed and made available to the researchers online. In addition, we present an analysis of testing and confirmed cases trends for different countries. We also present our online dashboard developed to monitor the progress of the virus through testing metrics and genomic variability analysis. We believe that our work will be crucial in monitoring the progress of SARS-CoV-2 in the attempt to end the pandemic.

## Supplementary Information

**Additional file 1:** **Table S1**. The table provides a listing of all the resources used to collect missing historical testing data points. It consists of 7 columns: Country/State, First Data Point, Last Data Point, Language, Data Type, Test Type reported and the Source reference. The “First Data Point” and “Last Data Point” columns indicate the date of the first and last manually collected data points, respectively. The “Language” column indicates the original language of the resource. The “Data Type” column indicates the format of the data (API: application programming interface, Infographic: uploaded data that gets overridden daily, Daily reports, News reports, Graphs and Machine readable datasets). The “Test Type Reported” column indicates the method used to test for SARS-CoV-2: PCR, serological or unspecified. The “Source” provides the URL to the resource used.

## Data Availability

A dynamically updated version of our dataset can be downloaded from github (https://github.com/KhazenLab/covid19-data) in CSV format. For the historical testing data statistics, each row represents a single day in a country or state/province. The fields are: CountryProv—Name of country or province in which the tests are reported. In cases of provinces, this field is a concatenation of country name with province name with a dash, e.g. “Australia—Queensland”. date—date on which the tests were done, in yyyy-mm-dd format. total_cumul.all—Cumulative number of tests done. total_cumul.source—Name of the source from which this row is obtained. Its value can be one of “owid”, “covidtracking”, “lau”, “wiki”, “worldometers”. interpolated—Yes/No field, “no” being for raw data, “yes” being for dates on which the number of tests is obtained from interpolation/extension of raw data. The genomic dataset summarizes the mutations found in each sample. Each row corresponds to an identified mutation in a sample and the columns correspond to the following: country—specifies the country the sample with identified mutation belongs to. date—indicates the collection date of the sample in a year-week format (i.e. 2020-3). mutation—position, reference nucleotide, query nucleotide. gene—the name of the gene in which the mutation is detected. consequence—the consequence type of the mutation. Mutation consequences can vary from (i) modifier such as upstream or downstream gene, (ii) low impact effect like synonymous mutation, (iii) moderate impact such as missense, and (iv) high impact such as frameshift, start or stop lost or gained. Finally, a mutation can be a splice region variant that can be of a low or moderate impact.

## Abbreviations

SARS-Cov-2: Sever acute respiratory syndrome coronavirus 2
Covid19: Coronavirus disease of 2019
OWID: Our World in Data
JHU: Johns Hopkins University
CTP: Covid Tracking Project (CTP)
LAU: Lebanese American University
API: Application Programming Interface
PCR: Polymerase chain reaction
SNP: Single nucleotide polymorphism
